# The Emerging Paradigm of Leadership for Future: The Use of Authentic Leadership to Lead Innovation in VUCA Environment

**DOI:** 10.3389/fpsyg.2021.759241

**Published:** 2021-11-23

**Authors:** Muhammad Mumtaz Khan, Syed Saad Ahmed, Essa Khan

**Affiliations:** Business Studies Department, Bahria Business School, Bahria University, Karachi, Pakistan

**Keywords:** authentic leadership, leader–member exchange, identification with the leader, VUCA, innovative work behavior

## Abstract

This study explicated the mediating role of leader–member exchange (LMX) and identification with the leader linking authentic leadership and innovative work behavior. The data were collected from the three sectors of the service industry. The final sample size obtained was 347. The data were collected both through the Google form and pen-filled questionnaires. SPSS was used to compute demographic profiles and conduct a hierarchal regression, while Smart-PLS was used for evaluating the constructs for their psychometric properties and testing the structural relations as proposed in the model. This study found LMX and identification with the leader to mediate between authentic leadership and the innovative work behavior of the employees.

## Introduction

The changing market space along with increased competition has created a new business paradigm in which competitive advantage does not last for too long. To describe the prevailing business environment characterized by volatility, uncertainty, complexity, and ambiguity, a new term volatile, uncertain, complex, and ambiguous (VUCA) has been introduced. VUCA is a game changer as it has pushed market leaders from their lead position to a peripheral status. With such a destabilizing ability of VUCA, the firms are hard-pressed to adjust themselves to ensure their survival and growth in the 21st century (Joshi et al., 2017). Successful adjustments appear in the form of innovative products and services (Nylén and Holmström, 2015) that in turn give a competitive advantage to the organization (Frishammar et al., 2019). Though the cure in the shape of adjustment and the wholesome result in the form of ensured survival and growth appears to be in a simple relation, the path to this adjustment is not so simple. Unlike the manufacturing economy where the top-led strategy ensured performance, the knowledge economy arguably abhors the idea of top-led performance (Mládková, 2012; Mládková et al., 2015). In the knowledge economy, employees play a pivotal role (Dess and Picken, 2000; Drucker, 2006). Equipped with knowledge and experience, they work as the source of ideas (Davenport, 2013). With this enhanced status of employees, leaders need to adjust themselves. Instead of command and control, the leaders require to play a facilitating role (Dess and Picken, 2000). The ideas of transformational and charismatic leader, well-suited for the manufacturing economy, do not hold much effect in the knowledge economy. The VUCA environment, because of its chaos, does not permit the use of a strategy driven by an individual or a group at the top (Livingston, 2014). The VUCA environment, instead, compels an enterprise to harness all of its cognitive resources to come up with innovative ideas fast (Chawla and Lenka, 2018). So, instead of idealized influence and charisma, the need of the hour is authenticity and humbleness (Millar et al., 2018), as in VUCA, it is not the pursuit of innovation driven through the idealized influence or charism of the leader, but the fast pursuit of innovation (Friedman, 2007; Watanabe et al., 2011), managed through authenticity.

To outpace the competitors, a leader needs to conflate ideas from multiple sources both internal and external (Dess and Picken, 2000). Though the use of ideas from multiple sources is quite useful for decision-making, it may give an unwanted signal to the subordinates as the leader is required to change the decisions with emerging clarity. The frequent change in decisions may frame the leader to be either weak or lack in integrity. In either cases, the loss of morale for the firm is high. Employees with such a negative perception of their leader may not be motivated to conceive and implement their innovative ideas (Javed et al., 2018). With the resulting ebb in an innovative effort, a firm cannot survive and grow in a VUCA environment (Millar et al., 2018). Neither slowing down the pace of decision-making nor the demotivation of the employees is productive for the organization even though both occur in tandem. The current study, instead of accepting the resulting distrust in a leader to be the byproduct of required adjustment to the emerging clarity, proposes a leadership style that at least reduces or at most eliminates the distrust emanating from the speedily changing decisions.

Authentic leadership has been in ascendance since the scandals of Enron and WorldCom. The deviation of a leader at the top proved to be apocalyptical for the said organizations washing thousands of jobs and wasting shareholders’ wealth. Since then authentic leadership along with other leadership strains like humble leadership, ethical leadership, and servant leadership is becoming more prominent. Authentic leadership is found to positively influence the performance of employees. At the employee level, authentic leadership is found to increase employees’ performance (Ribeiro et al., 2018; Hadian Nasab and Afshari, 2019), creativity (Xu et al., 2017; Zeb et al., 2019; Imam et al., 2020), and innovative work behavior (Laguna et al., 2019; Grošelj et al., 2021). With such an overarching influence, authentic leadership is being proposed to be a solution to the trust deficit emanating from the required changes to the decisions taken by the leadership of the organization. Authentic leaders, working for collective interests, can coalesce the subordinates around them (Steffens et al., 2016). The suitability of authentic leadership to spur employee innovative work behavior in a VUCA environment is being explained with three reasons. First, authentic leadership instills the thought of a collective goal for which all need to be authentic (Lyubovnikova et al., 2017). Sticking with the less optimal decisions and resisting a change go against the collective goal of the firm. Such behavior on part of a leader to take popular decisions, instead of optimal, is against the spirit of authentic leadership. Therefore, firms working in a VUCA environment are well served by leaders who readily accept their mistakes and change their decisions. Second, authentic leaders work for the collective wellbeing of all employees (Rahimnia and Sharifirad, 2015). When employees see their leaders showing a genuine concern for them, they begin to identify themselves with their leaders and readily come up with new ideas and work for their implementation despite frequent changes brought by the leadership. Finally, authentic leadership, through its clear communication, informs the followers about the final goal (Northouse, 2015). Additionally, they make employees realize that the final goal is something the whole organization seeks to attain while strategies are the tools to attain the goal. So, if the situation demands, the organization must comply by readily changing the existing strategy.

Apart from exploring the role of authentic leadership in affecting the innovative work behavior of the employees in organizations working in a VUCA environment, the current study adds to the lacuna in the existing literature. Though authentic leadership has been studied for its role to spur employee innovative work behavior (Grošelj et al., 2021), yet the mediating mechanism linking the two still requires further exploration. To this date, the relationship between authentic leadership and innovative work behavior has been studied using employee emotions (Zhou et al., 2014), personal initiative and work engagement (Laguna et al., 2019), and psychological capital (Purwanto et al., 2021) as mediators. The current study intends to explore the mediating role played by leader–member exchange (LMX) and identification with the leader. In doing so, the current study tests the social exchange theory (SET) whereby the favor extended by one party is returned by the favored party (Blau, 1964). The relationship quality (LMX) partly grows because of favor extended by the leader. This study, through the lens of SET, proposes that authentic leadership stretches out favor to the followers resulting in a high-quality relation. Motivated by this high-quality relation, employees indulge in different extra-role behavior such as innovative work behavior. Moreover, using identification with the leader as a linking mechanism relating authentic leadership and innovative work behavior, the current study is able to test the relevance of the social identity theory (Ashforth and Mael, 1989) in relation to authentic leadership.

The current study has these two objectives to accomplish. One, this study intends to unfurl the mediating role of LMX connecting authentic leadership to innovative work behavior. Two, this study unfurls a mediating path between authentic leadership and innovative work behavior passing through identification with the leader.

## Literature Review

### Authentic Leadership

Self-awareness reveals the true hidden self to an individual. More the people recognize their real selves, the more authentic they will become. According to Avolio et al. (2004), authentic leaders know how they think and behave; and how others perceive them based on their being aware of their own and others’ values, knowledge, and capabilities. Moreover, authentic leaders show hopefulness, confidence, optimism, resilience, and high moral character (Avolio et al., 2004). The actions of authentic leaders emanate from their values and conviction (Luthans and Avolio, 2003). Authentic leaders gel themselves with their followers by winning their trust and respect through the incorporation of followers’ input in decision-making and building collaborative networks with the followers (Avolio et al., 2004; Alilyyani et al., 2018). In short, authenticity in leadership is knowing, accepting, and remaining true to oneself (Avolio and Mhatre, 2011). The four key components of authentic leadership are balanced processing, relational transparency, internalized moral perspective, and self-awareness (Walumbwa et al., 2008).

The balanced processing component of authentic leadership means that instead of considering oneself to be the ultimate holder of wisdom, an authentic leader invites colleagues, and followers, for their input before arriving at the final decision (Chaudhary and Panda, 2018). Relational transparency, the second component of authentic leadership, refers to leaders’ open sharing of information, thoughts and feelings with followers (Wang et al., 2014). Internalized moral perspective, the third component of authentic leadership, is the internal form of self-regulation that guides people to make their decisions and indulge in behavior that is consistent with internalized moral values (Walumbwa et al., 2011; Crawford et al., 2020). Self-awareness among people matures when they realize who they are at the deepest level (Northouse, 2015). Leaders with self-awareness know their strengths and weaknesses and the goals they have in their lives (Ilies et al., 2005). Being clear about oneself truly provides people with a strong anchor for their decisions (Gardner et al., 2005).

An increasing number of studies point to the importance being given to authentic leadership in academia. There are studies relating authentic leadership to the work attitude of the employees. For instance, authentic leadership is found to foster organizational commitment (Leroy et al., 2012; Gatling et al., 2016), work engagement (Karam et al., 2017; Oh et al., 2018), and job satisfaction of the employees. Additionally, authentic leadership is found to positively influence employee performance (Ribeiro et al., 2018), proactive work behavior (Hu et al., 2018; Zhang et al., 2018), creativity (Černe et al., 2013; Chaudhary and Panda, 2018), and innovative work behavior (Grošelj et al., 2021).

### Identification With Leader

With the rise of relation-based leadership, the idea of subordinates identifying themselves with their leader is gaining currency. Kark et al. (2003) defined identification with the leader as the belief about the leader becomes self-defining. Hobman et al. (2011) defined identification with the leader as a self-categorizing process in which the subordinates strive to define themselves in the attributes of the leader, work for the gain of the leader, and experience connection with the leader. Identification with the leader is a construct that is mostly used either as a mediator (Miao et al., 2012; Gu et al., 2015; Jyoti and Bhau, 2015) or as a moderator (Wang and Rode, 2010) in leadership research.

### Leader–Member Exchange

Taking the relational view of the organization, the body of the organization can be regarded as the sum of relations. When these relationships are wholesome, the ensuing organization will also be a productive one. Conversely, an organization, with weak or negative relations among its members, will no doubt prove to be counter-productive. The quality of relationship between the leader and the subordinate, because of its ability to affect employee performance (Reb et al., 2019), has attained a valued status in organizational studies. The quality of the relationship between the leader and the subordinate is known as LMX (Graen and Uhl-Bien, 1995). With the high-quality relationship between the leader and follower, here being termed as high LMX, leader, and follower establish a relationship that is characterized by mutual trust and respect. The effectiveness of leadership, as per the lens of LMX, is obtained when the leader–follower dyad can have a mature relationship (Graen and Uhl-Bien, 1995). When there is high LMX, employees regard their leader to be attentive to their needs, accessible, and open for discussion and value their opinion (Liden and Graen, 1980; Martin et al., 2018). At the employee level, employees maintaining high-level LMX with their leader are found to be having a high commitment to the organization (Abu Bakar et al., 2009) and are productive (Reb et al., 2019) and innovative (Khalili, 2018; Mascareño et al., 2020).

### Innovative Work Behavior

Realizing the pivotal role played by the innovative performance of the firm, firms want all of their employees to pursue innovation. This all pursued innovations are different from the innovations that were pursued by the genius few. Innovative work behavior is a multistage process (de Jong and den Hartog, 2010). In the first stage, an idea is generated while at the second stage it is implemented (Devloo et al., 2015). Scott and Bruce (1994) broke innovative work behavior (IWB) into three stages. The stages, of course not sequential, are idea generation, idea promotion, and idea implementation. IWB is not limited to one specific area; it spans over innovation in product, process, and procedure. Consequently, it does not remain the handiwork of a genius few focusing on product design (West and Farr, 1990; Scott and Bruce, 1994). Now, an employee who is assigned to perform a routine process can think of process or procedural changes to indulge in IWB. IWB is found to positively influence the overall organizational attempt to pursue innovative performance (Fu et al., 2015; Sanz-Valle and Jiménez-Jiménez, 2018). Similarly, IWB promises positive effects for the employees. IWB is found to improve employees’ job satisfaction (West and Anderson, 1996) and performance (Leong and Rasli, 2014; Schuh et al., 2018).

### Authentic Leadership and Identification With the Leader

The strength of the relationship between the leader and follower is beneficial for the organization (Niu et al., 2018). The frequent interaction between leaders and followers is to learn about each other and is consequently adjusted to accommodate. Leaders, in this mutual relation, work from the point of strength; therefore, followers are influenced to adjust more as compared to the leaders. Identification with the leader is one such modification on part of the followers in which followers are inclined to share the values and beliefs of the leader and are ready to change their self-concept to enhance closeness to the ideas and beliefs of the leader (Kark et al., 2003). The three paths leading to identification with a person described by Ashforth et al. (2016) are: threat reducing; opportunity enhancing; and closeness enhancing. Considering authentic leadership, the path of threat reduction does not hold as authentic leader abhors indulging in office politics to favor someone (Kiersch and Byrne, 2015). Authentic leaders, guided by the highly internalized moral perspectives (Avolio et al., 2004), indulge in fair and honest dealings. As a result, followers hold their leaders in high esteem and tend to identify themselves with their leaders. Additionally, authentic leaders involve subordinates in decision-making (Walumbwa et al., 2008), as subordinates find themselves valued and respected, they not only increase their efforts to develop closeness with their leaders but also identify themselves with their leaders to avail more opportunities to prove their worth and to grow. Finally, the integral part of authentic leader’s self-concept is self-awareness (Walumbwa et al., 2011). Authentic leaders are not only well aware of their limitations but are also open to accepting others with their limitations. When employees are integrated in this way, the followers show a willingness to identify with their leaders. In short, a positive perception of the leader holds the followers in awe, and they are more likely to relate themselves with their leaders (Wang and Howell, 2012).

On the top of it, the relationship between authentic leadership and identification with the leader is also empirically verified (Pastor Álvarez et al., 2019). So, based on identity theory and the empirical evidence, the following hypothesis is formed.

H1: Authentic leadership is related to employee identification with the leader.

### Authentic Leadership and Leader–Member Exchange

Being an exchange process, high-quality LMX takes time to mature (Graen and Uhl-Bien, 1995). The high LMX quality ensures trust- and respect-based mutual relationship between the leader and the follower (Graen and Uhl-Bien, 1995; Scandura and Pellegrini, 2008). The initial relationship between the leader and the follower is mainly determined by the job description. With time, building on positive experiences, the relationship between the two moves from the scripted to the felt one and the employee is ready to go beyond what is described in the job description (Murphy and Ensher, 1999). Both leaders and followers bring in their resources for exchange and subsequently monitor the response of the transacting party to decide either to improve the relationship or not (Thompson et al., 2018). For instance, giving an added exposure to the subordinate and subsequently gauging the performance, the leader decides whether to repose trust in the follower. In case, the employee performs as expected or exceeds the expectation, the relationship quality will enhance. On the other hand, taking the initiative to enhance the relationship quality, subordinate volunteers perform a task. The resulting appreciation from the leader gives a hint to the employee to further invest in the relation. Apart from the individual exchanges, employees also strengthen or weaken the relationship with the leader based on their overall persona. Fair and transparent dealing with other employees proposes another clue to the subordinates to improve LMX quality with their leader (Mahsud et al., 2010; Tumasjan and Strobel, 2010).

Authentic leadership both through its dealing and through its overall image has the potential to invigorate relationships with the employees. By involving all in decision-making through its balanced processing (Avolio and Mhatre, 2011), authentic leadership gives a signal that it values all. Moreover, authentic leaders, through relational transparency (Gardner et al., 2005), maintain a good relationship with all the followers as they do not hold back information from their subordinates. Finally, self-awareness of the authentic leaders make them aware of their own weaknesses (Northouse, 2015). Consequently, authentic leaders are open to accepting the shortcomings of their subordinates. Working with leaders who involve the subordinates in decision-making, openly share information, ignore their mistakes, and honestly deal with them attracts the followers toward their leader, and resultantly the employees strive to establish strong relations with their leaders. Additionally, guided by an internalized moral perspective, authentic leaders establish themselves as honest individuals. Apart from the aforementioned reasoning, there is empirical evidence pointing to a relationship between authentic leadership and LMX (Lee, 2019). The established repute draws the followers toward their leaders and consequently, they intend to strengthen the relationship with their leaders. In the light of the above reasoning, the following hypothesis is formed.

H2: Authentic leadership is related to LMX.

### Identification With the Leader and Innovative Work Behavior

Innovative work behavior is an extra-role performance. Unlike the in-role performance that is affected by the ability of the employee, extra-role performance is affected by the motivation of the employees (Saether, 2019). The willingness of the employees to indulge in IWB is also affected by their motivation (Amabile and Kramer, 2011). Though the study conducted by Zhu et al. (2013) found identification with the leader to be negatively related to employee innovation (Miao et al., 2012), the current study purports identification with the leader to have a positive influence on innovative work behavior. When employees consider themselves to be related to their leaders, they internalize leaders’ values and goals (Kark and Shamir, 2013). Consequently, followers regard the extra-role behavior to be a bridge between leaders’ goals and they readily indulge in such behaviors as they are committed to the leaders’ goals (Wang and Howell, 2012). As leaders in the knowledge economy regard innovative work behavior to be important for the survival and growth of the organization (Khan et al., 2021) and constantly promote it among employees (Afsar and Umrani, 2019); therefore, the employees who positively identify themselves with their leaders will tend to pursue innovative work behavior. Though no study has explored the relationship between identification with the leader and innovative work behavior, there is a study that found identification with the leader to affect employee creativity (Gu et al., 2015). Guided by the provided reasoning and empirical evidence, the following hypothesis can be formed.

H3: Identification with the leader is positively related to innovative work behavior.

### Leader–Member Exchange and Innovative Work Behavior

Innovative work behavior has two major facets; the introduction of a creative idea and its implementation (Scott and Bruce, 1994). The creation of an innovative idea pertains to the use of cognitive processes (Smith and Ward, 2012). The associated risk of failure of the creative idea deters the employees to come up with an innovative idea (de Jong and den Hartog, 2010; Afsar et al., 2020). Faced with such a risky enterprise, the high relation quality between the leader and the follower works as a support for the employees. High-quality relation between the two gives employees the confidence to pursue a risk-infested process of introducing an innovative idea (Pathki et al., 2020). Furthermore, another part of innovative work behavior is to muster required support for the implementation of an idea (Scott and Bruce, 1994). High-quality relation between the leader and the follower turns out to be useful to achieve this end. High-quality LMX gives employees to share their idea with the leaders (Botero and Van Dyne, 2009) who have positional prowess to help in implementing the innovative idea. Additionally, coworkers, responding to the close connection between the employee and the leader, positively responds to the employee’s efforts for mustering support for the implementation of the innovative idea.

Contrary to the abovementioned reasoning in support of the relationship between LMX and innovative work behavior, the empirical evidence suggests otherwise. The study conducted by Agarwal et al. (2012) found the relationship between the two to be non-significant. Despite the contrary empirical evidence, we propose the following hypothesis.

H4: LMX is related to innovative work behavior.

### Mediating Role of Leader–Member Exchange

Employees, due to the inherent riskiness of IWB, do not pursue it unless they feel that they are supported by their leadership (Afsar and Umrani, 2019). Authentic leaders can provide this needed support by establishing high-quality relationships with the subordinates (Xu et al., 2017). When employees find their leaders seeking their input in decision-making and do not hold back any information from them; they begin to feel connected to their leaders. From the perspective of SET (Blau, 1964), a leader’s initiative of involving the subordinates in decision-making and upfront sharing of information with them work as a favor. Employees, in turn responding to the extended favor, take an initiative to indulge in pro-organizational behavior (Zou et al., 2015), i.e., innovative work behavior. Moreover, authentic leaders’ self-awareness (Walumbwa et al., 2008) makes leaders realize that very much like them, subordinates also make mistakes and by considering their mistakes to be a genuine effort to help the organization, they can be encouraged to take more initiatives to indulge in IWB. The leaders’ acceptance of their vulnerability and extending the same benefit to others succeed in binding the follower with their leader. Accepting followers’ shortcomings binds them in a high-quality relationship with the leader because followers regard this accommodating behavior to be a favor (Farson and Keyes, 2002). In return, followers show proclivity to extend the favor by indulging in pro-organizational behavior such as innovative work behavior. In light of the abovementioned theoretical reasoning, the following hypothesis is formed.

H5: LMX mediates the relationship between authentic leadership and innovative work behavior.

### Mediating Role of Identification With Leader

As discussed earlier, authentic leadership through its balanced processing internalized a moral perspective, and relational transparency can influence the followers to identify with them. Moreover, followers, once having identified with their leaders, tend to hold their leaders’ goals to be their own and strive for their realization (Kark and Shamir, 2013). Authentic leaders are influential in affecting followers’ relational identity, and the leader-centered identification motivates the employees to effort to attain leaders’ goals. Therefore, the following hypothesis is formed.

H6: Identification with the leader mediates the relation between authentic leadership and innovative work behavior.

**FIGURE 1:**
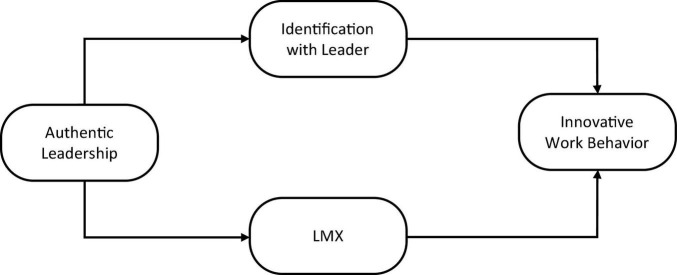
Conceptual framework.

The overall model is given in Figure 1.

## Methodology

### Sampling and Data Collection

The data were collected from employees working at firms operating in a VUCA environment. Employees working in healthcare, information technology (IT), and higher education are in a VUCA environment. Additionally, the employees working in the healthcare industry have been in high VUCA because of COVID-19. For this study, employees were approached. They were initially enquired whether they were employed in healthcare, IT, or higher education. Those who were from these sectors were requested to participate in this study. The respondents were assured of their anonymity and confidentiality of their data. Of the 425 approached employees, 395 respondents agreed to fill the questionnaire. Those who could fill the questionnaire online were directed to use a link to fill the questionnaire online. The respondents who found it hard to fill the questionnaire online were provided with a hardcopy of the questionnaire. Of the 395 filled questionnaires, 347 were found to be useful after discarding the ones with disengaged or missing responses.

Table 1 contains the profile of the respondents. Among 347 respondents, 57.3% (*n* = 199) were men while the remaining 42.7% (*n* = 148) were women. Apart from 4.6% (*n* = 16) of the respondents having intermediate (12 years of formal schooling), the rest were highly qualified. Among the respondents, 32.6% (*n* = 113) had a bachelor degree. A whopping 59.9% (*n* = 208) had a master degree, while a minor portion 2.9% (*n* = 10) was PhD. As mentioned above, the respondents were from the three different sectors of the service industry. The majority of the respondents, 42.9% (*n* = 149) to be exact were from higher education. Of the remaining respondents, 32.3% (*n* = 112) was from IT and 24.8% (*n* = 86) was from the healthcare sector.

**TABLE 1:** Respondents’ profile.

Variable	Values	*n* (347)	
Gender			
	Male	57.3%	
	Female	42.7%	
Qualification			
	Intermediate	4.6%	
	Bachelor	32.6%	
	Master	59.9%	
	PhD	2.9%	
Sector Working in			
	Healthcare	24.8%	
	Higher Education	42.9%	
	Information Technology	32.3%	
		Mean	SD
Age		30.910	8.558
Overall experience		7.631	5.475
Experience in the current field		5.463	5.475
Experience in the current organization		3.901	3.852

The average of the respondents was 31 years with a SD of 8.5 years as shown in Table 1. Additionally, the respondents on average had an experience of 7.6 years with a SD of 5.47 years. Similarly, Table 1 shows the average experience of the employees in the current industry being 5.46 years and the average number of years spent with the current employer being 3.9 years.

### Measure

#### Authentic Leadership

Authentic leadership was measured through the scale developed by Walumbwa et al. (2008). The scale has 16 items that were measured on a 7-point Likert scale where 1 denoted strongly disagree and 7 manifested strongly agree. One of the representative items of the scale was, “My leader is willing to admit mistakes when they are made.”

#### Identification With the Leader

To measure identification with the leader, the scale developed by Kark et al. (2003) was used. The scale has eight items. One of the representative items of the scale is “I identify strongly with my leader.” The scale was measured on a 7-point Likert scale where 1 was used to denote strong disagreement with the statement and 7 was used to present strong agreement with the statement.

#### Leader–Member Exchange

Leader–member exchange was measured through the scale developed by Graen and Uhl-Bien (1995). The scale has seven items. One of the representative items is, “How well does your leader recognize your potential.” All the items were measured on a 7-point Likert scale. The scale responses varied from one item to another item.

#### Innovative Work Behavior

Innovative work behavior was gauged through the scale developed by de Jong and den Hartog (2010). The multi-item Likert scale developed by those authors has 10 items (de Jong and den Hartog, 2010). A representative item for the scale is, “How often do you generate original solutions for problems?” All the items were measured on a 7-point Likert scale where 1 was intended to denote never and 7 was used to present always.

### Data Analysis Strategy

Data were analyzed by using Smart-PLS and SPSS. Using Smart-PLS, the data were checked for their reliability and validity. After ascertaining the quality of data, a structural model was run using 5,000 iterations. To further improve the rigor of this study, the latent variables created in Smart-PLS were imported into SPSS along with the demographic variables to apply a hierarchical regression.

## Findings

### Measurement Model

The testing of the structural model is preceded by the evaluation of the measurement model. To ascertain the appropriateness of measurement model reliability, the convergent validity and discriminant validity were evaluated. The results are being discussed one by one. First of all, Cronbach’s alpha (α) and composite reliability (CR) were used to evaluate the constructs for their inter-item consistency. As shown in Table 2, all the constructs had α and CR more than 0.7, the minimum acceptable limit for both the criteria (Chin, 2010; Taber, 2018; Hair et al., 2019). The minimum of α (0.927) and CR (0.942) were found for LMX. Second, to ascertain the convergent validity, average variance extracted (AVE) and factor loadings were used. For construct level convergent validity, the construct is required to have an AVE of at least 0.5 (Henseler et al., 2015; Hair et al., 2017). The values for AVE for all the constructs were in excess of this minimum threshold value. In this research, the lowest value of AVE was found for authentic leadership that was 0.650 as shown in Table 2 confirming construct-level convergent validity. Additionally, to confirm item-level convergent validity, the item of each construct should have a loading of at least 0.7 (Hair et al., 2014). As shown in Table 2, all the items have a loading of more than 0.7 on their respective construct, except for the first item of 0.696 innovative work behavior yet the item was retained as it was not eroding the AVE of IWB too much to skid below 0.5, the minimum acceptable limit of AVE. Finally, to ascertain the discriminant validity of the constructs used in this study, hetero-trait mono-trait (HTMT) ratios for all pairs of constructs were computed. The constructs are declared to have discriminant validity when the HTMT ratio is less than 0.85. The less stringent rule allows the HTMT ratio to go up to 0.9 (Henseler et al., 2015). As shown in Table 3, all pairs of HTMT are found to be less than 0.85 except for the HTMT ratio for authentic leadership and IWB in that case it was 0.889. Though the value is more than 0.85, yet it falls in the lenient acceptable range of HTMT ratios.

**TABLE 2:** Reliability and validity.

		Loadings						
Variable	Items	AL	IWB	IWL	LMX	Alpha	CR	AVE
Authentic leadership	AL1	0.766				0.964	0.967	0.650
	AL10	0.780						
	AL11	0.799						
	AL12	0.863						
	AL13	0.770						
	AL14	0.822						
	AL15	0.811						
	AL16	0.808						
	AL2	0.791						
	AL3	0.760						
	AL4	0.765						
	AL5	0.848						
	AL6	0.803						
	AL7	0.752						
	AL8	0.858						
	AL9	0.887						
Innovative work behavior	IWB1		0.696			0.958	0.964	0.730
	IWB10		0.899					
	IWB2		0.798					
	IWB3		0.843					
	IWB4		0.848					
	IWB5		0.879					
	IWB6		0.869					
	IWB7		0.895					
	IWB8		0.899					
	IWB9		0.895					
Identification with leader	IWL1			0.830		0.945	0.954	0.721
	IWL2			0.836				
	IWL3			0.852				
	IWL4			0.874				
	IWL5			0.852				
	IWL6			0.871				
	IWL7			0.869				
	IWL8			0.808				
LMX	LMX1				0.768	0.927	0.942	0.698
	LMX2				0.878			
	LMX3				0.830			
	LMX4				0.878			
	LMX5				0.776			
	LMX6				0.871			
	LMX7				0.840			

**TABLE 3:** Descriptive statistics and hetero-trait mono-trait (HTMT) ratios.

			Correlation			HTMT Ratio		
Variable	Mean	SD	AL	IWB	IWL	AL	IWB	IWL
Authentic Leadership (AL)	4.735	1.396	1					
Innovative Work Behavior (IWB)	4.710	1.558	0.564**	1		0.653		
Identification with Leader (IWL)	5.096	1.203	0.892**	0.515**	1	0.889	0.680	
Leader–Member Exchange (LMX)	4.351	1.360	0.739**	0.432**	0.735**	0.721	0.609	0.706

**Significant at 1% significance level.

### Structural Model

Before constructing a structural model, correlations among the constructs and variance inflation factors (VIFs) are evaluated. The inter-construct correlations are found to be either moderate or strong correlations. The correlation values are strong enough to support the development of a structural model. However, there is a need to evaluate the VIFs for the constructs used in the model before running a structural model (Tarka, 2018). The constructs are declared to be safe from an extreme level of multicollinearity if the VIF values are less than 5. The results depicted in Table 4 show the VIF values to be less than 5, thus ensuring the absence of an extreme level of multicollinearity among the constructs.

**TABLE 4:** Variance inflation factor (VIF).

Variable	AL	IWB	IWL
Authentic leadership (AL)			
Innovative work behavior (IWB)	1		
Identification with leader (IWL)	1	1.803	
Leader–member exchange (LMX)	1	1.803	1

This study had six hypotheses to test. Table 5 contains the results for the hypotheses that are being discussed one by one. The first hypothesis proposed a relationship between authentic leadership and identification with the leader. The obtained results supported the relationship between authentic leadership and identification with the leader (β = 0.888, *p* = 0.000). The second hypothesis, in this study, conjectured the relation between authentic leadership and LMX. The result presented in Table 5 shows authentic leadership and LMX to be significantly related (β = 0.687, *p* = 0.000). The third hypothesis states the relationship between identification with the leader and innovative work behavior. The values of path coefficient and the value of *p* presented in Table 5 show the proposed relationship to be significant (β = 0.483, *p* = 0.000). The last of the direct hypotheses, purporting a relationship between LMX and innovative work behavior, was also turned out to be significant (β = 0.253, *p* = 0.000).

**TABLE 5:** Structural model.

Relation	β	SE	*t*-test	*p*-value
Authentic leadership → identification with leader	0.888	0.013	70.662	0.000
Authentic leadership → LMX	0.687	0.033	20.989	0.000
Identification with leader → innovative work behavior	0.483	0.059	8.132	0.000
LMX → innovative work behavior	0.253	0.064	3.961	0.000
Authentic leadership → LMX → innovative work behavior	0.174	0.045	3.892	0.000
Authentic leadership → identification with leader → innovative work behavior	0.429	0.055	7.872	0.000

**FIGURE 2:**
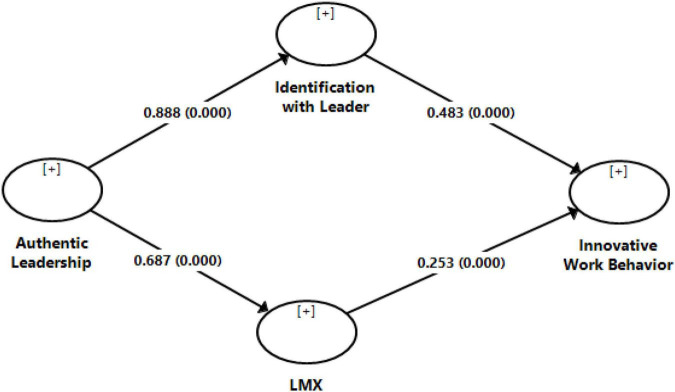
An estimated structural model.

Along with the abovementioned direct relationships, this study also proposed a mediating path relating authentic leadership with innovative work behavior. The first proposed linking process between authentic leadership and innovative work behavior was passed through LMX. The results, as shown in Table 5, find LMX to be a significant mediator (β = 0.174, *p* = 0.000). The second linking mechanism between authentic leadership and innovative work behavior, proposed in this study, went through identification with the leader. The results, as shown in Table 5, support our conjecture of user identification with the leader to be a mediator between authentic leadership and innovative work behavior (β = 0.429, *p* = 0.000). The results of path analysis are pictorially shown in Figure 2.

### Additional Analysis

To unfurl the role of control variables and to evaluate the incremental role of each variable in the model, a hierarchal regression was run. The results given in Table 6 indicate that the three control variables, namely gender, age, and qualification of the respondents, do not significantly contribute to the explanatory power of any of the models. The maximum explanation was found for Model 1 where identification with the leader was a dependent variable (Δ*F* = 1.901, *p* = 0.129). We thus find that the control variables do not significantly contribute to the explanatory power of any of the endogenous variables, namely identification with the leader, LMX, and innovative work behavior.

**TABLE 6:** Hierarchical regression model.

		Model (dependent variable)		
Step	Independent variable	Model 1 (identification with leader)	Model 2 (LMX)	Model 3 (innovative work behavior)
1	Gender	−0.081	−0.005	0.009
	Age	−0.095	−0.061	−0.029
	Qualification	0.108	0.014	0.114
	R^2^	0.016	0.003	0.011
	ΔR^2^	0.016	0.003	0.011
	ΔF (Sig.)	1.901 (0.129)	0.342 (0.795)	1.240 (0.295)
2	Gender	−0.021	0.042	
	Age	−0.037	−0.015	
	Qualification	0.073	−0.013	
	Authentic leadership	0.884**	0.689**	
	R^2^	0.793	0.475	
	ΔR^2^	0.776	0.472	
	ΔF (Sig.)	1,280.671 (0.000)	307.551 (0.000)	
3	Gender			0.062
	Age			0.033
	Qualification			0.043
	Identification with leader			0.654**
	R^2^			0.431
	ΔR^2^			0.421
	ΔF (Sig.)			252.884 (0.000)
4	Gender			0.049
	Age			0.032
	Qualification			0.058
	Identification with leader			0.480**
	LMX			0.256**
	R^2^			0.467
	ΔR^2^			0.036
	ΔF (Sig.)			23.095 (0.000)

**Significant at 1% significance level.

In the second step of Model 1, the introduction of authentic leadership along with the control variables significantly increased the explanation of identification with the leader (Δ*F* = 1,280.671, *p* = 0.000). Similarly, in the second step of Model 2 in which the endogenous variable was LMX, the inclusion of authentic leadership along with the control variables significantly improved the explanatory power of the model (Δ*F* = 307.551, *p* = 0.000). For Model 3, the inclusion of identification with the leader increased the explanation of innovative work behavior significantly as depicted by the results shown in step 3 of Model 3 (Δ*F* = 252.884, *p* = 0.000). The inclusion of LMX along with identification with the leader and the control variables improved the explanation of innovative work behavior significantly (Δ*F* = 23.095, *p* = 0.000).

## Discussion

This study began with the six hypotheses. First, this study explored the relationship between authentic leadership and identification with the leader. The findings of this study supported the relationship between authentic leadership and innovative work behavior. The finding corroborates the earlier finding made by Pastor Álvarez et al. (2019). The second hypothesis of this study, claiming a relationship between authentic leadership and LMX, was also found to be significant that corroborates the earlier findings made by Lee (2019). The current study also attempted to ascertain the relationship between identification with the leader and innovative work behavior. The results found the relationship to be statistically significant, thus corroborating the similar relation established by Yang et al. (2019) between identification with the leader and employee creativity. The last direct relationship tested is the relationship between LMX and employees’ innovative work behavior. The obtained results found LMX to have a positive relationship with the innovative work behavior of the employees. However, this is against the earlier findings made by Agarwal et al. (2012) who concluded LMX and innovative work behavior to be having a non-significant relationship. The difference in findings can be attributed to the difference in the population under study as the current study obtained the data from knowledge workers while the study conducted by Agarwal et al. (2012) collected the data from service sector employees. Another reason that can be forwarded is the time elapsed since the study done by Agarwal et al. (2012).

### Theoretical Contribution

This study makes four important contributions to the existing body of knowledge. First, adding to the theory of identification (Turner et al., 1979; Ashforth and Mael, 1989; Ashforth et al., 2016), the current study found identification with the leader to be related to another positive work outcome that is innovative work behavior. Individuals tend to identify themselves with social groups that add to their positive image (Turner et al., 1979). Leaders with authenticity are one of such groups that may attract the individuals to identify with (Javaid et al., 2015). Identification with leaders coalesces employees with their leader, and they regard the leadership’s goals to be theirs, thus pursuing them with a similar zeal. Second, the study added to the theory of LMX. LMX, the quality of the relationship between leader and employee (Graen and Uhl-Bien, 1995; Schriesheim et al., 1999), was ascertained to have a positive relationship with the innovative work behavior of the employees. This finding adds to the accepted proposition of LMX to have a positive impact on pro-organizational outcomes (Schyns, 2006; Harris et al., 2011; Teng et al., 2020). Third, the study added to the existing body of knowledge of identification theory. The study found that identification with the leader works as a mediator between authentic leadership and the innovative work behavior of the employees. This study informs that identification with the leader is affected by the authenticity of the leader. The finding has practical relevance as relatively more assertive knowledge workers value character, openness, and they are being accepted in decision-making. Finally, this study adds to the body of knowledge related to the SET (Blau, 1964) as it finds the favorable attitude shown by the leaders forges a high-quality relationship between leaders and employees that leads to positive organizational behavior such as innovative work behavior.

### Managerial Implications

The need for the current study was triggered by the increasing VUCAness of the environment. The yesteryears rapidity of staling rate of products and services along with an ever-expanding commanding role of employees, as knowledge workers are more actively getting the central stage, is joined by COVID-19 to add to the complications of the organizations. In such a fuzzy environment, the change in way of leadership is correctly called for. Employees in the current era are not inclined to buy the idealized influence and charisma of the leader. They, instead, expect their leaders to show authenticity. The need for authenticity is further necessitated by leaders’ compulsion to adjust their decisions as more information reduces ambiguity. The use of idealized influence and charisma do not hold much chance to succeed as both of them compel the leaders to stick to the past decision while the need of the hour is to be ready to adjust with the emerging clarity. The organizations need to encourage the managers to practice authentic leadership, so the managers cannot only adjust to the emerging changes but also win the support of their followers.

The two mediating paths between authentic leadership and innovative work behavior have multiple managerial implications. First, managers need to understand the importance of having a strong bond with subordinates. By building a strong relationship with employees, managers can motivate employees to pursue innovative work behavior. Organizations can provide opportunities to managers and employees to frequently interact with each other. These opportunities can both be formal job interactions and informal off-the-job interactions such as picnics and other outings. Similarly, authenticity has an instrumental role in affecting employees to identify with their leaders. With Enron’s and WorldCom’s scandals still etched into your memory, firms can foster a positive image of leadership through the show of authenticity. Organizations, along with claims to authenticity, need to establish a congruence between their words and actions, so employees are encouraged to identify themselves with their organizations. Organizations need to encourage their managers to be more ostensive in their show of authenticity. Practices like participative decision-making process, making informed decisions, and accepting mistakes of others as well-intended efforts to serve a common cause will be some of the positive practices that can motivate employees to identify themselves with their leaders that will subsequently lead to their pro-organization behavior. One of such behaviors is the employees’ proclivity to indulge in innovative work behavior.

### Limitations and Future Direction

To improve the generalizability of this study, the data have been collected from the three different sectors, namely IT, healthcare, and higher education. Besides this, this study has got some limitations. Collecting data from a single source instead of a dyad makes the result susceptible to a common method error (Podsakoff et al., 2003). Moreover, the same problem may arise as the data were collected at a single point of time (Podsakoff et al., 2003) and an independent variable is not allowed to have enough time to register its effect (Cox, 1992; Warner, 2017). To make up for these limitations, future researchers are suggested to use employee–manager dyads for collection. Similarly, to allow the independent variable, authentic leadership, to register its effect, the cause and effect needs to be measured at two different times. By using the two waves of data collection, future researchers can circumnavigate the common method error arising from single time data collection. Apart from these methodological limitations, no study has the potential to include all the linking mechanisms and boundary conditions. The current study proposes the use of meaningfulness and work flow as the mediators linking authentic leadership and innovative work behavior. This suggestion is in line with the authentic leadership theory that suggests positive emotions work to improve employee performance (Northouse, 2015). Similarly, on part of boundary conditions, the relationship between authentic leadership and innovative work behavior can be tested while using organizational structure and considering employees learning focus as moderators.

## Data Availability Statement

The raw data supporting the conclusions of this article will be made available by the authors, without undue reservation.

## Author Contributions

All authors listed have made a substantial, direct and intellectual contribution to the work, and approved it for publication.

## Conflict of Interest

The authors declare that the research was conducted in the absence of any commercial or financial relationships that could be construed as a potential conflict of interest.

## Publisher’s Note

All claims expressed in this article are solely those of the authors and do not necessarily represent those of their affiliated organizations, or those of the publisher, the editors and the reviewers. Any product that may be evaluated in this article, or claim that may be made by its manufacturer, is not guaranteed or endorsed by the publisher.
